# Accuracy of Ultra-Fast Low-Field MRI (0.55 T) for Lung Nodule Detection with Ultra-Short Echo Time Sequences

**DOI:** 10.3390/tomography11120132

**Published:** 2025-11-26

**Authors:** Maximilian Hinsen, Armin Michael Nagel, Nadine Bayerl, Hans-Peter Fautz, Thomas Benkert, Matthias Stefan May, Michael Uder, Rafael Heiss

**Affiliations:** 1 Institute of Radiology, University Hospital Erlangen, Friedrich-Alexander-Universität (FAU) Erlangen-Nürnberg, 91054 Erlangen, Germany; 2 Division of Medical Physics in Radiology, German Cancer Research Center, 69120 Heidelberg, Germany; 3 Imaging Science Institute Erlangen, Siemens Healthineers, 91054 Erlangen, Germany; 4 Research & Clinical Translation, Magnetic Resonance, Siemens Healthineers AG, 91052 Erlangen, Germany

**Keywords:** lung neoplasms, magnetic resonance imaging, mass screening, lung, early detection of cancer

## Abstract

Background: This study aims to evaluate the detection rate of modern low-field Magnetic Resonance Imaging (MRI) with single-breath-hold ultra-short echo time (UTE) sequences for detection of lung nodules and to subsequently correlate nodule size with the measurements obtained by computed tomography (CT). Methods: From January to April 2021, a prospective clinical trial at a single academic center evaluated ultra-fast low-field MRI (0.55 Tesla) for detecting pulmonary nodules. Patients with known nodules underwent MRI and CT on the same day. The primary aim was to assess nodule detection with UTE sequences, with secondary analysis focused on mean nodule diameter correlation with CT. Mann–Whitney U tests, Spearman’s correlation, and Bland–Altman analysis were applied to the data. A *p*-value < 0.05 was considered statistically significant. Results: A total of 30 patients (mean age, 59 years ± 13 [standard deviation], 17 female and 13 male) were enrolled. In a blinded MRI and CT imaging analysis, 21.6% (176/814) of lung nodules were correctly identified on MRI. The detection rate was 91.2% (52/57) for lung nodules with a mean diameter of ≥6–<8 mm and 100% for those ≥8 mm (31/31). Pulmonary nodules < 4 mm were detected in 4.3% (24/562) and nodules of ≥4–<6 mm were detected in 42.1% (69/164). The mean difference in size measurements between MRI and CT was 0.43 ± 1.1 mm, with Spearman’s correlation coefficient of r = 0.85 (*p* < 0.001). Conclusions: Modern ultra-fast low-field MRI with UTE sequences is capable of detecting pulmonary nodules (≥6 mm) and demonstrates a strong correlation with CT in measuring nodule size. However, it was not effective in detecting nodules smaller than 4 mm, suggesting that MRI-based nodule detection methods require further optimization before clinical implementation.

## Simple Summary

Ultra-short echo time magnetic resonance imaging allows visualization of the lung parenchyma, which is traditionally challenging because of rapid signal decay. In this study, we evaluated the detection rates of a breath-hold lung magnetic resonance imaging protocol for identifying pulmonary nodules compared with computed tomography. Although magnetic resonance imaging demonstrated reliable detection of larger nodules, it was less effective for small ones. This study highlights both the potential and the current limitations of modern low-field magnetic resonance imaging with ultra-short echo time sequences for lung nodule detection.

## 1. Introduction

Lung nodules are a common radiological finding that can be caused by a variety of reasons, ranging from benign granulomas and scarring to the early stages of primary lung malignancies and metastases. Reliably detecting and characterizing these lesions is an important part of chest radiology. Because of its high spatial resolution, which results in high detection rates, and its fast, cost-effective examination times, computed tomography (CT) is the reference standard for lung imaging and nodule detection. However, it involves not insignificant radiation exposure.

Early detection and reliable monitoring of lung nodules are critical for staging patients with cancer of other primaries and for the early detection of lung cancer, which remains the leading cause of cancer-related mortality worldwide. Previous international studies have shown that patient outcomes can be improved by early diagnosis of lung carcinomas or metastases [1,2,3]. To enable timely diagnosis of early malignant stages, lung cancer screening programs have been implemented in the United States and are on the raise in various other countries like Germany, and France [1,4,5,6].

Recent technical advances in the field of low-field Magnetic Resonance Imaging (MRI) offer promising opportunities for clinical radiation-free imaging by combining the benefits of inherent reduction in susceptibility artifacts with state-of-the-art software and hardware [7,8,9,10]. Several recently published studies have already emphasized the clinical applicability of modern low-field MRI for different body parts, including functional and morphological lung imaging [7,11,12,13,14].

A previous study at 1.5 Tesla (T) suggested that MRI-based lung cancer screening, according to the Lung-RADS recommendations, is feasible without missing lung cancer at any stages [15]. These promising results were even outperformed by recent study results using a modern low-field MRI at 0.55 T [16]. One of the significant drawbacks of these studies was that they used dedicated lung MRI protocols with breath-gated and motion artifact-reduced sequences. Therefore, they had long acquisition times between 7:12 min and 16:39 min respectively [15,16]. Reducing examination time could be a critical improvement, making lung MRI a viable, radiation-free alternative to CT for detecting and monitoring lung nodules. As an alternative, lung MRI with ultra-short echo time (UTE) sequences could be used to reduce signal decay in the lung tissue and to examine the entire lung in only a few seconds [17,18,19].

According to current guidelines from the American College of Radiology, lesion size and its size dynamics remain the most important factors in determining the therapeutic approach for pulmonary lesions [20]. If fast low-field MRI with UTE sequences were to become an alternative to CT, it would have to show high detection rates for suspicious nodules. Moreover, size measurements should be reliable for rational recommendations to the clinician.

Based on these considerations, this study aimed to compare the detection rate of ultra-fast low-field MRI for pulmonary nodules with CT as the reference standard and to correlate size measurements between the two modalities to assess its capability for lung nodule detection and follow-up examinations.

## 2. Materials and Methods

### 2.1. Ethical Approval

Approval for this prospective study was granted by the local ethics committee of the Friedrich-Alexander-Universität (FAU) Erlangen-Nürnberg (approval number 483_20 B, 16 December 2020). Prior to inclusion in the study, written informed consent was obtained from each participant.

### 2.2. Study Design

A cross-sectional investigator-initiated trial was conducted between 15 January 2021, and 6 April 2021, at a single academic medical center to evaluate the detection rate of ultra-fast low-field MRI for pulmonary nodule identification. Patients with previously confirmed lung nodules on CT and scheduled for a follow-up chest CT were consecutively enrolled. The inclusion criteria were (1) known lung nodules based on the radiology information system, (2) a medical indication for CT examination, and (3) the ability to give informed consent. Study exclusion criteria were (1) MRI contraindication (e.g., cardiac pacemaker), (2) pregnancy, and (3) claustrophobia. A medical doctor contacted patients who met the inclusion criteria one week prior to their scheduled CT examination and invited them to voluntarily participate in the study. Each patient underwent both a lung MRI and a CT examination on the same day.

### 2.3. Patient Selection

**Figure 1:**
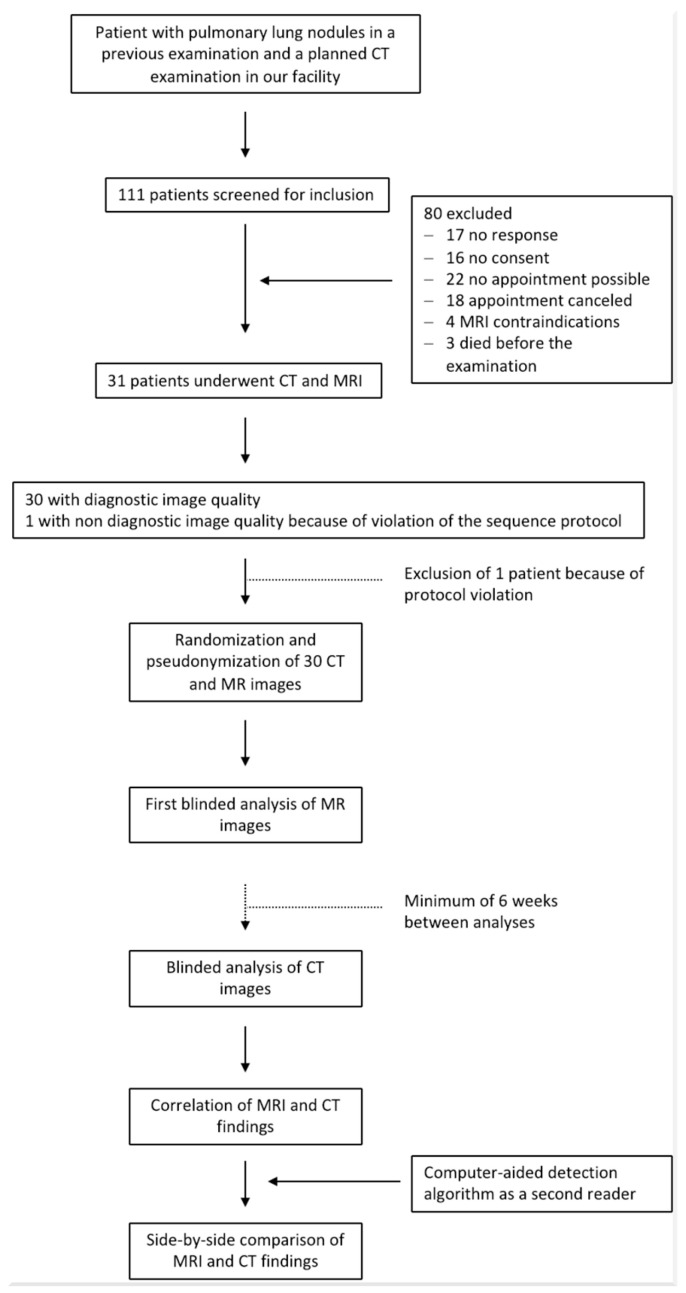
Flowchart illustrating the study process.

Initially, the eligibility of 111 consecutive patients presenting with lung nodules and scheduled for a chest CT scan was assessed. Participation was not possible for 40 patients due to scheduling conflicts, 17 patients did not respond to the invitation, 16 declined to participate, 4 were excluded because of MRI contraindications, and 3 patients died before they could participate. In one case, incorrect image parameters were used for image acquisition, which violated the study protocol and resulted in nondiagnostic image quality. Therefore, the images of this patient were excluded from further evaluation. Finally, ultra-fast lung MRI was included of 30 patients (mean age, 59 ± 13 [SD]; 17 female, 13 male). Due to the identical inclusion criteria, the cohort of the current study partially overlaps with that of a previously published study, as the same inclusion criteria and time overlap meant that the 111 consecutive patients screened were a subset of the 210 patients included in the earlier work [16]. The presented data of the current study have not been published elsewhere as it deals with a separate analyzing process of a different MRI protocol. Figure 1 demonstrates the study design and Table 1 presents the characteristics of the participants.

### 2.4. Computed Tomography

The CT examinations were acquired in the clinical routine using three different CT scanners. Most were performed on 128-slice CT scanners (25 patients on a SOMATOM go.Top) and 2 on a SOMATOM X.cite system, Siemens Healthcare, Erlangen, Germany). Three examinations were performed on a 192-slice Dual Source CT scanner (SOMATOM Force, Siemens Healthcare, Erlangen, Germany). The reconstruction parameters are presented in Table 2.

### 2.5. Magnetic Resonance Imaging

Ultra-fast lung MRI was performed using a 0.55 T MRI system (MAGNETOM Free.Max, Siemens Healthcare, Erlangen, Germany). Patients were examined in a head-first supine position with arms down. Phased-array receiver coils included a 9-channel spine array, a 6-channel flex coil, and a 12-channel head/neck array. The last coil was used because previous tests revealed better image quality of the cranial lung fields. Two anisotropic ultra-short echo time (UTE) protocols were set up to offer an ultra-fast MRI of the lungs with image correlation in at least two planes. Transversal and coronal orientation respectively were used for primary acquisition. Acquisitions with this research application was performed during breath-holding using a 3D stack-of-spirals sampling pattern with variable TE encoding [21]. Spiral imaging was performed in-plane, while conventional Cartesian sampling was applied for the partition direction [22]. The MRI sequence parameters of our protocol are presented in Table 3.

### 2.6. Imaging Evaluation

A radiologist (M.H.) randomized and pseudonymized the CT and MR images. After this, the blinded datasets were passed to the reader for separate evaluation on a 3D post-processing console (Syngo.Via, Siemens Healthcare GmbH, Erlangen, Germany). The images were assessed on a diagnostic monitor (EIZO RX660, Hakusan, Japan) by a radiologist with 9 years of experience in cross-sectional imaging (R.H.). The assessment was performed and analyzed using a two-step approach, as previously published [16]. First, MR images were evaluated, and each detected lung lesion was matched between the transversal and coronal planes to avoid false positive results. The reader measured the nodule size for the longest and orthogonal diameters to one decimal place in the transversal plane of the MRI. Every lung nodule (solid, part solid, ground glass) was evaluated. There was no analysis of other lung pathologies such as consolidation, ground glass, reticulation, or atelectasis. To minimize recall bias, the evaluation of the CT images was conducted six weeks later, using computer-assisted three-dimensional (3D) multiplanar reformations and maximum intensity projections. All images were also subjected to computer-aided detection (CAD; syngo. CT Lung CAD, Siemens Healthcare GmbH, Erlangen, Germany) as an additional evaluation to enhance the detection rate of the reader [23,24]. The reader was blinded to personal and clinical information during the image interpretation. Nodule mean diameters were calculated to one decimal place.

For the assessment of the detection rates, the detected MRI and CT lung nodules were compared in a one-to-one fashion on a dual-monitor workstation. The MRI detected nodules were paired with the corresponding nodules identified on CT imaging for subsequent statistical comparison.

Furthermore, each missed nodule was thoroughly reviewed to assess whether it could be retrospectively identified on MRI through direct comparison with CT images.

### 2.7. Statistical Analysis

Statistical analysis was performed using the STATA 17.0 software package (StataCorp, College Station, TX, USA). CT measurements were retrospectively performed for nodules not detected in the blinded CT analysis but identified on MRI. Detection rates were calculated for following subgroups, which were defined based on Lung-RADS Assessment Categories 2022 [20]: solid, part solid, and ground glass nodules, <4 mm, ≥4–<6 mm, ≥6–<8 mm, ≥8–<15 mm, ≥15 mm, for the blinded analysis, the side-by-side comparison. The nodule margin was categorized as smooth, lobulated or spiculated, and the location was categorized as central, perifissural or subpleural. The MRI detection rate was calculated as the proportion of nodules detected on MRI relative to those identified on CT. False-positive MRI results were reported separately.

Given that normal distribution could not be assumed, Spearman’s rank correlation coefficient was employed to evaluate the correlation between nodule sizes measured by CT and MRI. Bland–Altman analysis was performed to assess the agreement between the results. The Mann–Whitney U test was used to compare nominal variables. Adjusted *p*-values are presented, and *p*-values below 0.05 were considered statistically significant across all analyses.

## 3. Results

### 3.1. Lesion Detection

**Figure 2:**
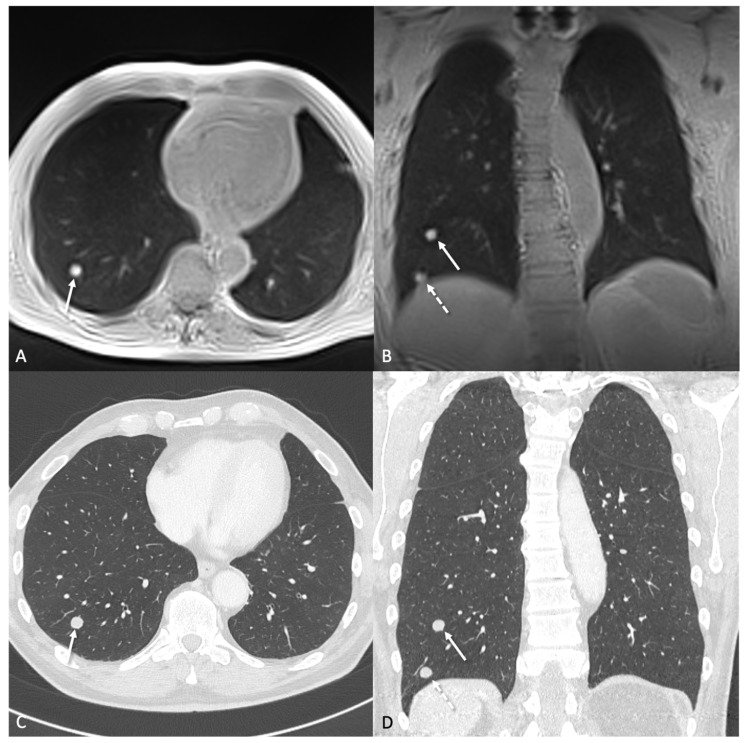
A 76-year-old patient with transitional cell carcinoma. The transversal (**A**) and coronal (**B**) ultra-short echo time (UTE) sequence show a 11.3 × 9.8 mm metastasis (arrow) in the right lower lobe. The coronal planes (**B**,**D**) additionally show another 11.9 × 9.5 mm pulmonary metastasis next to the right diaphragm (dashed arrow). (**C**,**D**) show the corresponding CT at 1 mm slice thickness obtained on the same day.

**Figure 3:**
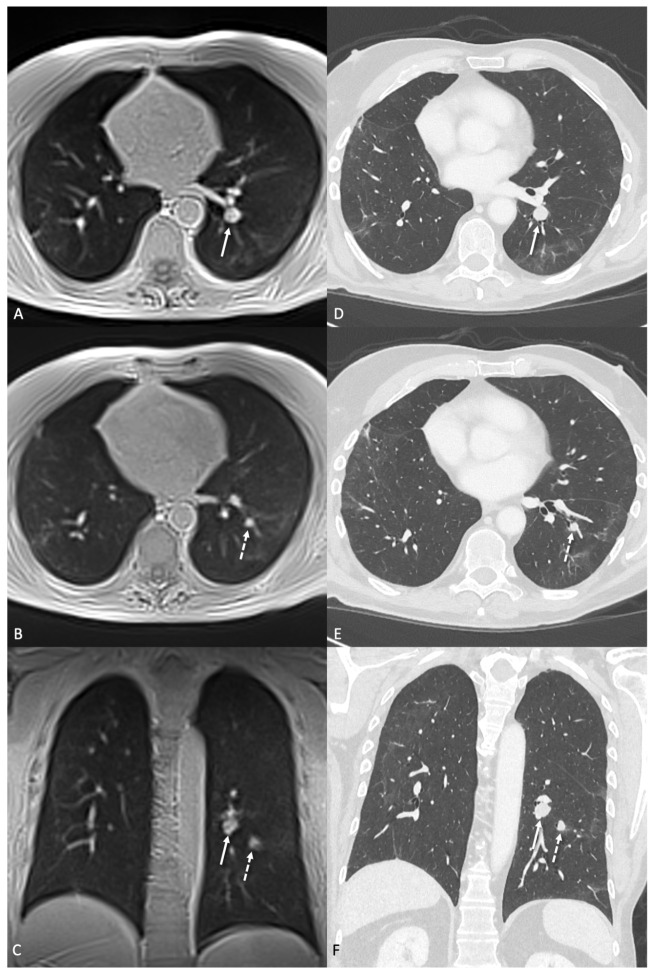
Two metastases of a malignant melanoma in the left lower lobe. The bigger nodule (arrow) with 16.7 × 13.6 mm is next to the left hilus vessels but clearly visible in transversal (**A**) and coronal (**C**) ultra-short echo time (UTE) sequences. The second is located a bit more caudally and has a size of 11.2 × 7.4 mm (dashed arrow) but is also clearly visible on MRI (**B**,**C**). (**D**–**F**) show the corresponding CT images with 1 mm slice thickness.

**Figure 4:**
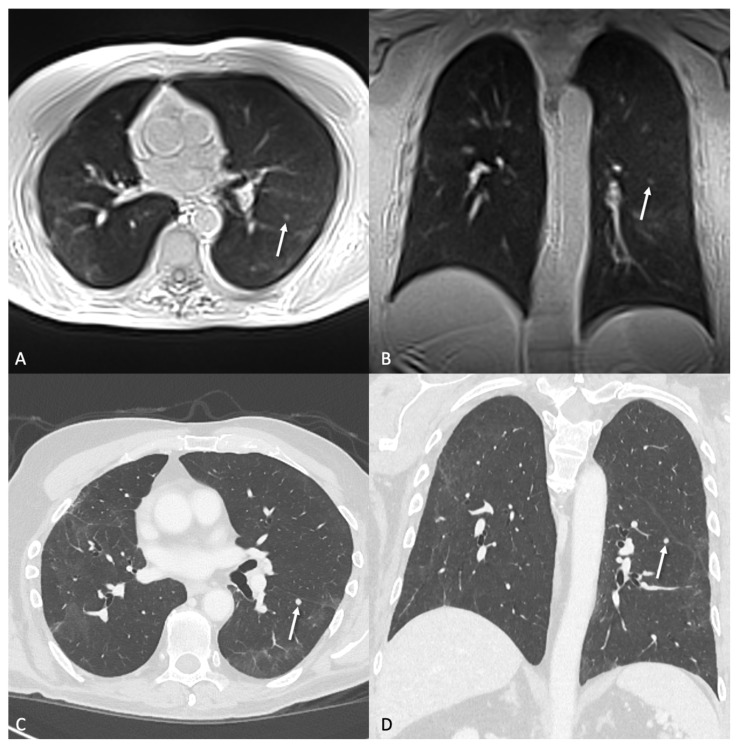
A 77-year-old patient with bipulmonary metastases of a malignant melanoma. The transversal (**A**) and coronal (**B**) ultra-short echo time (UTE) sequence clearly showed a 4.5 × 4.4 mm lung metastasis (arrow) in the left lower lobe. (**C**,**D**) depict the same nodule on CT with a 1 mm slice thickness, acquired on the same day.

In 30 CT examinations, a total of 814 lung nodules were identified, with a mean diameter of 3.8 ± 2.1 mm. All patients presented with at least one nodule, with an average of 27 nodules per patient. Figure 2, Figure 3 and Figure 4 illustrate examples of representative lung nodules.

Ultra-fast MRI successfully detected 176 nodules, while 638 nodules were missed, yielding an overall detection rate of 21.6%. Notably, all nodules with a diameter of ≥8 mm identified by CT were also detected by MRI (31 nodules, detection rate 100%). Nodules detected by MRI had a mean diameter of 6.4 ± 3 mm, which was significantly larger (*p* < 0.001) than the mean diameter of nodules missed by MRI (3.1 ± 1.0 mm). The smallest nodule detected by MRI measured 2.2 mm, while the largest was 19.5 mm in diameter. Of the 88 nodules with a diameter of ≥6 mm, 83 (94.3%) were accurately identified by MRI. In contrast, among nodules with a diameter of <4 mm, MRI detected only 24 out of 562 (4.3%).

**Figure 5:**
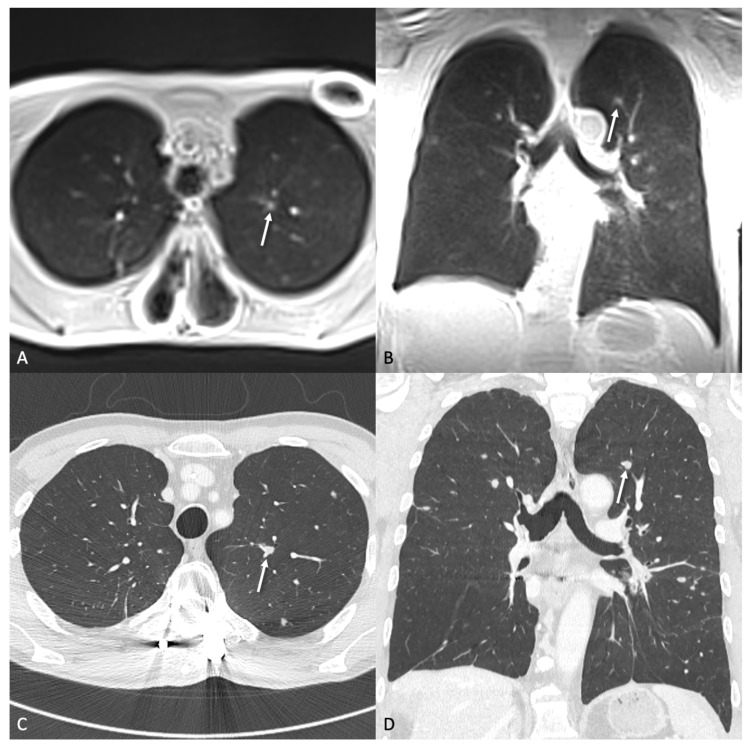
A nodule (arrow) with a size of 6.1 × 4.1 mm in the left upper lobe in a 61-year-old patient with colorectal carcinoma. The nodule was not detected in the blinded analysis of the MRI by the reader probably because of the adjacent lung vessels. In direct comparison with CT, it is possible to clearly identify the nodule in transversal (**A**) and coronal (**B**) ultra-short echo time (UTE) MRI compared to respective CT images (**C**,**D**).

Forty lung nodules (4.5 ± 0.8 mm) were missed in the initial blinded analysis of the MRI and were detected retrospectively in a side-by-side comparison with CT images. For 19 missed lung nodules, potential reasons were identified that prevented the detection. In 8 cases the nodules were near the pleural surface, in 5 cases perifissural, in 3 cases near other structures (1 next to lung vessels, 1 to dystelectasis, and 1 to another nodule), while 1 was a ground glass nodule, 1 was not visible in the coronal plane, and 1 was only visible in the coronal plane because of aliasing artifacts in the transversal plane. Figure 5 shows an example of a nodule missed in blinded analysis but visible in direct comparison to CT. Table 4 presents more data about the location and composition of the lung nodules in this study.

Missed nodules had a diameter of 0.9 to 7.7 mm with 347 of the 638 nodules (54.4%) having a diameter of ≤3 mm. The distribution of the remaining undetected lesions was as follows: 32.3% (206/638) were >3–≤4 mm, 9.7% (62/638) were >4–≤5 mm, 3.0% (19/638) were >5–≤6 mm, 0.5% (3/638) were >6–≤7 mm, and 0.2% (1/638) was >7–≤8 mm.

In MRI, 9 lesions were misinterpreted as lung nodules. Their mean size was 6.6 ± 2.3 mm (range 3.7–10.6 mm). In direct comparison to CT images, 3 turned out to be pleural lesions, 5 were pulmonary scars and 1 had no anatomic correlate. The latter lesion was located peribronchovascularly in close proximity to the pulmonary hilum.

Four ground glass nodules with a mean diameter of 4.3 ± 2.1 mm were detected in CT, with a detection rate of 50.0% (2/4) for MRI, and 7 part solid nodules with a mean diameter of 9.5 ± 3.6 mm were detected, with a resulting detection rate of 100% (7/7).

**Figure A1:**
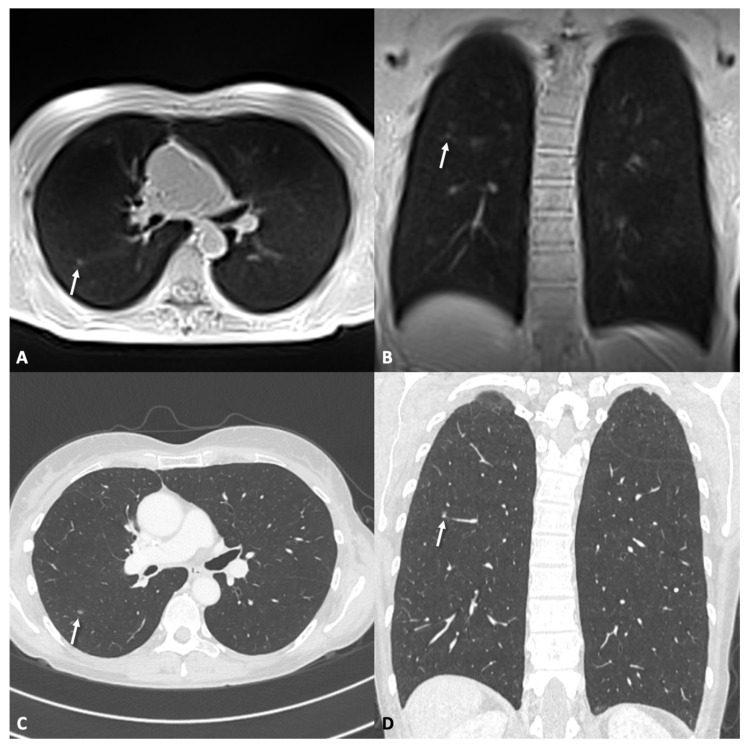
A 3.8 × 3.7 mm lung nodule (arrow) was detected in transversal (**A**) and coronal (**B**) lung MRI sequences with ultra-short echo-time (UTE) in the blinded analysis. Both reader and computer-aided detection (CAD) missed the nodule during the analysis of CT images, but it was clearly visible in side-by-side comparison. (**C**,**D**) show the corresponding CT with 1 mm slice thickness.

Two nodules (mean diameter of 6.2 ± 3.4 mm) were detected by UTE but missed by both the radiologist and CAD on CT (Figure A1).

### 3.2. Nodule Size Correlation

**Figure 6:**
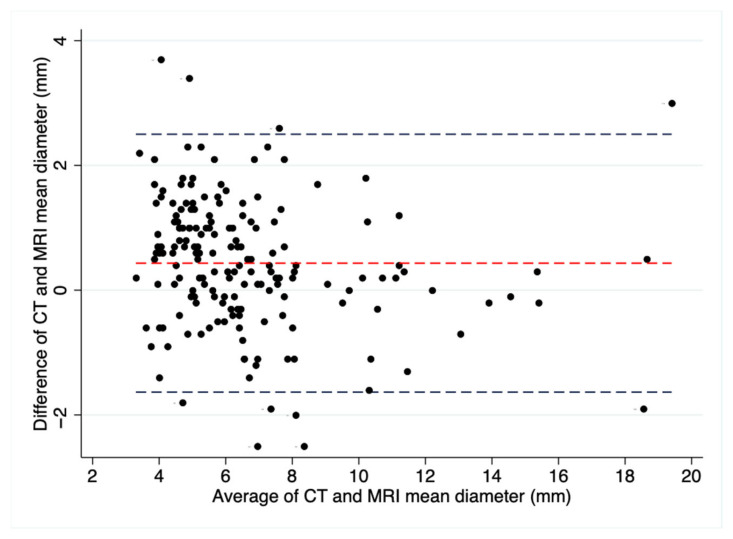
Bland–Altman plot illustrating the agreement in mean nodule diameter between CT and MRI (black dots). The mean difference between MRI and CT was 0.43 mm (red dashed line). A total of 10 of 176 nodules (5.7%) are outside the agreement limits. The 95% limits of agreement ranged from −1.63 mm to 2.5 mm (blue dashed lines). Mean nodule diameters measured on CT and MRI ranged from 3.3 mm to 19.4 mm.

A total of 176 nodules were identified in both MRI and CT, with a strong positive correlation observed between the mean nodule diameters measured by MRI and CT (r = 0.85, *p* < 0.001). The Bland–Altman plot illustrates a high level of agreement in mean diameter measurements between CT and MRI, showing a mean difference of 0.43 ± 1.1 mm (Figure 6).

## 4. Discussion

This study aimed to investigate whether ultra-fast, low-field MRI at 0.55 T using UTE could serve as a viable alternative to CT for the detection of suspicious pulmonary nodules. To address this, we conducted a prospective study involving 814 lung nodules in 30 patients. The findings demonstrated a high detection rate of 94.3% (83/88) for nodules measuring ≥6 mm, with all nodules ≥ 8 mm identified on MRI. Notably, the majority of missed nodules were ≤4 mm (84.3%, 538/638). MRI and CT measurements of nodule size were highly correlated (r = 0.85, *p* < 0.001), with a mean size difference of only 0.43 ± 1.1 mm.

Chest CT continues to serve as the reference standard for lung imaging due to its rapid scan time, wide availability, and high spatial resolution, advantages not matched by other imaging modalities. Recent studies have highlighted the potential of modern low-field MRI as a radiation-free alternative [7,13,14,25,26]. An essential part of chest imaging is the reliable detection of early lung cancer stages because of the high mortality of this condition in its advanced stages [1,27,28,29]. Recent studies reported high detection rates for lung nodule detection with MRI at different field strengths. A recent study using modern low-field MRI revealed an excellent detection rate of 100% for nodules ≥ 6 mm [16]. The high detection rates were enabled by breath-gated and artifact-reduced sequences, with a relatively long average examination time of up to 16:39 min. Another study demonstrated the image quality of self-gated 3D UTE sequences at 0.55 T with isotropic resolution, which was enabled by a combination of robust respiratory binning, trajectory correction, and concomitant field correction, with an acquisition time of 8.5 min [30]. A short examination time is critical to minimizing costs and maximizing availability, especially for screening examinations. Therefore, we tried to reduce the examination time as much as possible by employing breath-hold techniques.

For these reasons, a previous study examined single breath-hold UTE sequences at a 3 T field strength but could not prove sufficient accuracy for lung nodule detection [31]. According to its results, UTE under high-frequency non-invasive ventilation showed the best results with a detection rate of 35% for 66 nodules ≥ 4 mm and 50% for 32 nodules ≥ 6 mm [31]. In contrast, a recently published study reported that 107 of 149 lung nodules could be detected using UTE sequences at 3 T. The detection rate for the subgroup with nodules > 4 mm was even better at 90.2% (92/102) [32]. Another study compared the detection of lung nodules using breath-gated and single-breath-hold MRI with UTE sequences at 1.5 T, reporting detection rates of 75.2% to 77% for 165 nodules with a mean diameter of 7.6 mm [33]. We aimed to extend these promising findings for UTE sequences with those of modern low-field MRI for lung examinations. This approach enabled acquisition times under 20 s for each plane, making breath-gated imaging unnecessary. In addition, examinations can be performed in inspiration at full expansion of the lungs as recommended in CT. We acquired a transversal and a coronal plane to correlate imaging findings in two planes and to reduce false positive results. However, the short acquisition time led to a sparse utilization of the field of view in large patients. As a result, aliasing artifacts masked one nodule in the transversal plane of one examination.

The detection of perifissural and pleural nodules seemed to be especially challenging in our study. This could be due to susceptibility and Gibb’s artifacts, which affect the area adjacent to the pleura more strongly than in breath-gated sequences with additional artifact reduction techniques [34,35]. Even though this resulted in a lower detection rate, the clinical significance of these lung nodules is questionable, since perifissural and pleural lesions are mostly benign [36].

Our findings demonstrated an excellent detection rate of 100% (31/31) for lung nodules measuring ≥8 mm and a very high detection rate of 91.2% (52/57) for nodules measuring ≤ 6–<8 mm. However, these rates could not compete with those of a recently published study on low-field MRI at 0.55 T with breath-gated sequences. The authors of that study reported a 100% detection rate (126/126) for nodules ≥ 6 mm, and an 80% detection rate (159/200) for nodules ≥4–<6 mm [16]. In our present study, the detection rate was insufficient for nodules measuring ≥4–<6 mm. This limitation is particularly relevant for the screening of high-risk patients because based on the Lung-RADS recommendations they require shortened screening intervals for part-solid nodules starting at a size of 4 mm [20]. This is a critical drawback when considering UTE lung MRI for lung cancer screening, especially for part-solid nodules. Comparing our present results with those of previous studies that used breath-hold lung MRI with UTE sequences reveal mixed findings. While our approach outperformed one previous study [31], it lagged behind other results obtained at 1.5 T and 3 T. It is important to note that the nodules in our study were smaller (3.8 ± 2.1 mm) than those reported in previous studies at 1.5 T (7.6 ± 3.7 mm) and 3 T (11.8 ± 9.4 mm) [32,33].

Our study had several limitations. Firstly, the patient cohort was small and comprised individuals with different underlying diseases in advanced stages. Secondly, we were unable to compare our results with other field strengths or obtain histological information for the detected nodules. Due to ethical concerns, we could not include healthy controls, which prevented the calculation of specificity. Thirdly, evaluation was only performed by one reader, which could have affected the evaluation. However, previous studies indicate that using computer-aided detection as a second reader for lung nodule assessment outperforms conventional double reading [24]. Moreover, the study consists of only a small number of 4 ground-glass and 7 partially solid nodules, which limits the reliability of these subgroup results. We also did not use any acceleration techniques for our UTE sequences, which could provide an opportunity for improving image quality in the future.

To our knowledge, this is the first feasibility study to evaluate lung nodule detection using UTE sequences on a modern low-field MRI system. We showed that multiplanar lung imaging can be performed in under 40 s, achieving detection rates of 91.2% for nodules ≥ 6–8 mm and 100% for nodules ≥ 8 mm. On the other hand, the image quality is more dependent on the patient’s physical conditions than in breath-gated techniques and detection rates for smaller nodules are insufficient. Because this study included patients with severe medical conditions, the results cannot be directly applied to other groups, such as screening populations.

## 5. Conclusions

Ultra-fast 0.55-T MRI using UTE sequences shows promise for the assessment of larger pulmonary nodules, achieving high detection rates within very short acquisition times. However, its performance for smaller nodules remains insufficient, and image quality is more susceptible to patient-related factors than in breath-gated approaches. While ultra-fast UTE MRI has potential for future lung imaging applications, further technical refinement is required before it can be considered for broader clinical use, including lung cancer screening.

## Figures and Tables

**Table 1:** Participant Demographics and Clinical Characteristics.

Characteristics	Patients (N = 30)
**Mean Age—years**	59 ± 13
**Mean Weight—kg**	79.6 ± 21
**Mean Height—cm**	172.2 ± 8.7
**Sex—no. (%)**	
Female	17 (56.7)
Male	13 (43.3)
**Race or ethnic group—no. (%)**	
Mixed, other	1 (3.3)
White	29 (96.7)
**Disease (%)**	
Lung cancer	6 (20.0)
Other primary cancers	22 (73.3)
(post-)Infectious	2 (6.7)

Note—Percentages are given in parentheses. Continuous variables are expressed as mean ± SD.

**Table 2:** Computed tomography Examination and Reconstruction Parameters.

Parameter	Examination and Reconstruction Parameters		
Scanner	128-slice single source CT	192-slice dual source CT	128-slice single source CT
Tube voltage (kV)	90–140	100	120
Reference (mAs)	99	172	129
Filter	non	non	gold & tin
Matrix	512 × 512	512 × 512	512 × 512
Orientation	transversal	transversal	transversal
Slice thickness	1.0 mm	1.0 mm	1.0 mm
Reconstruction increment	0.7 mm	0.7 mm	0.7 mm
Reconstruction kernel	Br60	Br60	Br60
Window setting	Lung window	Lung window	Lung window
Window width (HU)	1700	1700	1700
Window level (HU)	−600	−600	−600

Abbreviations: Computed tomography (CT); kilovolt (kV); milliampere-seconds (mAs); millimeter (mm), Hounsfield units (HU).

**Table 3:** MRI ultra-short echo time (UTE) protocols.

Parameter	Transversal	Coronal
Slice thickness	3 mm	3 mm
In-plane resolution	2.1 × 2.1 mm^2^	2.1 × 2.1 mm^2^
Field of view (FOV)	480 × 480 mm^2^	480 × 480 mm^2^
Acquisition time	19 s	18 s
Repetition time (TR)	10 ms	9.5 ms
Echo time (TE)	0.27 ms	0.05 ms
RF excitation	Selective	Non-selective
Acceleration technique	None	None
Breath-hold	Inspiration	Inspiration

Abbreviations: Echo time (TE); Field of view (FOV); Magnetic resonance imaging (MRI); millimeter (mm); square millimeters (mm^2^); milliseconds (ms); seconds (s); Repetition time (TR); Radiofrequency (RF); Ultra-short echo time (UTE).

**Table 4:** Subgroups of lung nodules.

Mean Nodule Diameter	<4 mm	≥4–<6 mm	≥6–<8 mm	≥8–<15 mm	≥15 mm
Number of nodules	562	164	57	26	5
Nodules detected in MRI (%)	4.3% (24/562)	42.1% (69/164)	91.2% (52/57)	100% (26/26)	100% (5/5)
Nodules detected in direct comparison of CT and MRI (%)	6.4% (36/562)	59.2% (97/164)	91.2% (52/57)	100% (26/26)	100% (5/5)
Solid nodules detected on MRI (%)	4.3% (24/561)	41.6% (67/161)	90.6% (48/53)	100% (24/24)	100% (4/4)
Part solid detected on MRI (%)	0/0	100% (1/1)	100% (3/3)	100% (2/2)	100% (1/1)
Ground glass detected on MRI (%)	0/0	33.3% (1/3)	100% (1/1)	0/0	0/0
Smooth margin (%)	4.1% (23/557)	41.3% (59/143)	86.8% (33/38)	100% (13/13)	100% (2/2)
Lobulated margin (%)	20.0% (1/5)	50.0% (8/16)	100% (17/17)	100% (11/11)	100% (1/1)
Spiculation (%)	0	40.0% (2/5)	100% (2/2)	100% (2/2)	100% (2/2)
Central nodules (%)	4.9% (21/433)	43.1% (53/123)	94.7% (36/38)	100% (18/18)	100% (4/4)
Subpleural nodules (%)	3.8% (3/80)	46.2% (12/26)	82.4% (14/17)	100% (5/5)	100% (1/1)
Perifissural nodules (%)	0% (0/49)	26.7% (4/15)	100% (2/2)	100% (3/3)	0

Abbreviations: Computed tomography (CT); Magnetic resonance imaging (MRI); millimeter (mm).

## Data Availability

The data presented in this study are available on request from the corresponding author. This restriction is in place to protect the privacy of our patients and is in accordance with our ethical approval.

## Abbreviations

The following abbreviations are used in this manuscript:

CAD	Computer-aided detection
cm	Centimeter
CT	Computed tomography
DICOM	Digital Imaging and Communications in Medicine
FOV	Field of view
kg	Kilogram
kV	Kilovolt
HU	Hounsfield Unit(s)
mAs	Milliampere-seconds
mm	Millimeter
mm^2^	Square centimeters
MRI	Magnetic Resonance Imaging
ms	Milliseconds
RF	Radiofrequency
s	Seconds
SD	Standard Deviation
UTE	Ultra-short echo time
T	Tesla
TE	Echo time
TR	Repetition time
